# Stereological Estimates of Glutamatergic, GABAergic, and Cholinergic Neurons in the Pedunculopontine and Laterodorsal Tegmental Nuclei in the Rat

**DOI:** 10.3389/fnana.2018.00034

**Published:** 2018-05-11

**Authors:** Esther Luquin, Ibone Huerta, María S. Aymerich, Elisa Mengual

**Affiliations:** ^1^Division of Neurosciences, Center for Applied Medical Research (CIMA), University of Navarra, Pamplona, Spain; ^2^Department of Biochemistry and Genetics, School of Science, University of Navarra, Pamplona, Spain; ^3^Anatomy Department, School of Medicine, University of Navarra, Pamplona, Spain

**Keywords:** basal ganglia, GAD65, GAD67, Vglut2, gait, reward

## Abstract

The pedunculopontine tegmental nucleus (PPN) and laterodorsal tegmental nucleus (LDT) are functionally associated brainstem structures implicated in behavioral state control and sensorimotor integration. The PPN is also involved in gait and posture, while the LDT plays a role in reward. Both nuclei comprise characteristic cholinergic neurons intermingled with glutamatergic and GABAergic cells whose absolute numbers in the rat have been only partly established. Here we sought to determine the complete phenotypical profile of each nucleus to investigate potential differences between them. Counts were obtained using stereological methods after the simultaneous visualization of cholinergic and either glutamatergic or GABAergic cells. The two isoforms of glutamic acid decarboxylase (GAD), GAD65 and GAD67, were separately analyzed. Dual *in situ* hybridization revealed coexpression of GAD65 and GAD67 mRNAs in ∼90% of GAD-positive cells in both nuclei; thus, the estimated mean numbers of (1) cholinergic, (2) glutamatergic, and (3) GABAergic cells in PPN and LDT, respectively, were (1) 3,360 and 3,650; (2) 5,910 and 5,190; and (3) 4,439 and 7,599. These data reveal significant differences between PPN and LDT in their relative phenotypical composition, which may underlie some of the functional differences observed between them. The estimation of glutamatergic cells was significantly higher in the caudal PPN, supporting the reported functional rostrocaudal segregation in this nucleus. Finally, a small subset of cholinergic neurons (8% in PPN and 5% in LDT) also expressed the glutamatergic marker Vglut2, providing anatomical evidence for a potential corelease of transmitters at specific target areas.

## Introduction

The PPN and the LDT are two closely associated brainstem structures that jointly participate in a variety of functions such as behavioral state control, sensorimotor integration and reinforcement, and learning (Winn, 2006; Garcia-Rill et al., 2016; Mena-Segovia, 2016). At the same time, the PPN has been typically associated with gait control and posture in close association with the basal ganglia (Pahapill and Lozano, 2000; Mena-Segovia et al., 2004), being a potential therapeutic target for deep brain stimulation in Parkinson’s disease patients with severe gait alterations (Collomb-Clerc and Welter, 2015; Hamani et al., 2016). In turn, LDT appears to be particularly implicated in reward (Lammel et al., 2012, 2014; Kohlmeier, 2013; Redila et al., 2015; Steidl and Veverka, 2015; Steidl et al., 2017; but see also Winn, 2008 and Norton et al., 2011 regarding PPN involvement in reward).

Common functions of the PPN and LDT are anatomically supported by reciprocal connections, and also by broadly common afferent and efferent projections (Hallanger and Wainer, 1988; Mitani et al., 1988; Steriade et al., 1988; Semba and Fibiger, 1992; Charara and Parent, 1994; Motts and Schofield, 2009, 2010; Mellott et al., 2011). However, segregation of their respective output projections and/or regional complementarity at their target structures has also been reported (Woolf and Butcher, 1986; Hallanger et al., 1987; Hallanger and Wainer, 1988; Semba and Fibiger, 1992; Oakman et al., 1995; Kita and Kita, 2011).

At the cellular level, the PPN and LDT comprise a mixed population of cholinergic and non-cholinergic cells. While the cholinergic cell groups were characterized in the early eighties (Ch5 and Ch6, respectively; Mesulam et al., 1983), the non-cholinergic cells have been unambiguously identified only recently as GABAergic and glutamatergic using the mRNAs of the GABA synthetic enzyme GAD and the Vglut2 as specific markers, respectively (Boucetta and Jones, 2009; Mena-Segovia et al., 2009; Wang and Morales, 2009). While the phenotypical identification of PPN and LDT cell subpopulations represents an important step to understand the roles of these nuclei, the estimation of the total numbers of each subpopulation is also essential in order to establish potential differences in the connectivity and functions of each nucleus. The use of unbiased stereological methods of quantification has been generally accepted as the way to obtain an accurate measurement of neurons (Saper, 1996). Thus, the first goal of the present study was to estimate and compare the total numbers of each of the three neurochemical subpopulations in the PPN and LDT, using stereological methods. A previous elegant stereological study already estimated the total counts of cholinergic and GABAergic cells in the PPN (Mena-Segovia et al., 2009). Here, Immunohistochemistry against ChAT and ISH against either Vglut2 or GAD mRNA were used to identify the three phenotypes, respectively. A dual colorimetric method was used that enabled to distinctly visualize either cholinergic and glutamatergic cells, or cholinergic and either GAD67- or GAD65-positive cells, directly in a single section.

GABA is synthesized by two isoforms of GAD, GAD65 and GAD67. The constitutive cytosolic GAD67 appears to provide for the core GABA for inhibitory transmission, while the hydrophobic GAD65 – which is transiently activated – seems to synthesize GABA for high-frequency bursts to fine-tune GABAergic synaptic function (Tian et al., 1999; Patel et al., 2006). Interestingly, the mRNAs of the two isoforms are heterogeneously expressed in the diverse nuclei of the basal ganglia in rat (Mercugliano et al., 1992; Esclapez et al., 1994), and their expression is altered after experimental depletion of substantia nigra dopaminergic cells (Kincaid et al., 1992; Soghomonian and Chesselet, 1992; Delfs et al., 1995). Thus, we also sought to determine whether there were any differences between the PPN and LDT in their relative distribution of GAD isoforms, analyzing separately GAD65- and GAD67-positive subpopulations in both nuclei.

Finally, several studies have reported the presence of glutamate in PPN cholinergic cells in different species (Clements et al., 1991; Lavoie and Parent, 1994). In parallel, electrophysiological studies have identified substantial functional heterogeneity both within cholinergic and non-cholinergic neurons in the PPN (Steriade et al., 1990; Takakusaki et al., 1996, 1997; Mena-Segovia et al., 2008). In order to determine whether this heterogeneity might partly be due to the presence of specific cell subsets coexpressing neurotransmitters or neuromodulators, the second goal of our study was to analyze the potential coexpression of markers in the two nuclei. Potential dually labeled cells were analyzed and confirmed using dual or triple fluorescence immunolabeling.

## Materials and Methods

### Animals and Tissue Preparation

Eight adult male Wistar rats (250–350 g; Harlan, Barcelona, Spain) were used in this study. All experimental procedures were carried out in accordance with the guidelines of the National and European Council on the use of animals on research (RD 1201/2005 and 86/609/EEC). The experimental design was approved by the Ethical Committee for Animal Testing of the University of Navarra.

Rats were deeply anesthetized with a mixture of ketamine (150 mg/kg; Imalgene 500, Merial Laboratories, France), xylazine (20 mg/kg; Rompún 2%, Bayer Health Care, Spain), and atropine (0.1 mg/kg, i.p.; Atropina, Braun Medical SA, Barcelona, Spain), and transcardially perfused with 220 mL of saline Ringer’s solution followed by 500 mL of cold fixative containing 4% paraformaldehyde in 0.1 M PBS pH 7.4. The brains were then removed, postfixed in the same fixative solution at 4°C overnight and immersed in a cryoprotection solution containing 20% glycerin and 2% dimethylsulfoxide in 0.125 M PB pH 7.4 for 24 h, also at 4°C (Rosene et al., 1986). All solutions were treated with 0.1% of diethyl pyrocarbonate (DEPC, Sigma) and autoclaved prior to their use. After incubation for 1 h in 30% sucrose, the brains were sectioned in the coronal plane using a freezing microtome, and the 40-μm-thick sections serially collected in the same cryoprotection solution.

### Riboprobe Preparation

Sense and antisense riboprobes of rat GAD65, GAD67, and Vglut2 were transcribed as described previously (Erlander et al., 1991; Tillakaratne et al., 1992; Stornetta et al., 2002a,b). GAD65 and GAD67 plasmids were generously donated by Drs. A. J. Tobin and N. J. K. Tillakaratne (Department of Biology, University of California, Los Angeles, CA, United States), while the Vglut2 plasmid was kindly gifted by Drs. R. L. Stornetta and P. Guyenet (Department of Pharmacology, University of Virginia, Charlottesville, VA, United States).

Each plasmid (7 μg) was linearized in a solution with a final V of 30 μl for 4 h at 37°C and then transcribed with the appropriate RNA polymerases (Boehringer Mannheim, Mannheim, Germany) in order to synthesize either sense or antisense probes. The transcription mixture (20 μl) included 1 μg of template cDNA, 1× biotin-NTP labeling mix (Roche Diagnostics, GmbH Mannheim, Germany), or digoxigenin-NTP (Roche, Diagnostics, GmbH Mannheim, Germany), 1 U/μl inhibitor (Promega, Madison, WI, United States), and 1 U/μl of either T7, T3, or SP6 RNA polymerase (Roche) and was incubated for 2 h at 37°C. The template cDNA was digested with 10 U RNase-free DNAse for 30 min at 37°C, then the proteins were eliminated with 0.5 μg of proteinase K (Roche) for 15 min at 55°C, and finally proteinase K was inactivated for 5 min at 90°C. The riboprobes were then precipitated by adding 20 μl of TE-0.1% DEPC, 40 μl of 4 M ammonium acetate, and 200 μl of ethanol for at least 1 h at -20°C, recovered by centrifugation at 12,000 rpm for 30 min at 4°C, washed with 70% ethanol with H_2_O-0.1% DEPC, and finally resuspended in autoclaved H_2_O-0.1% DEPC.

### Dual Colorimetric Labeling of *in Situ* Hybridization and Immunocytochemistry

**FIGURE 1:**
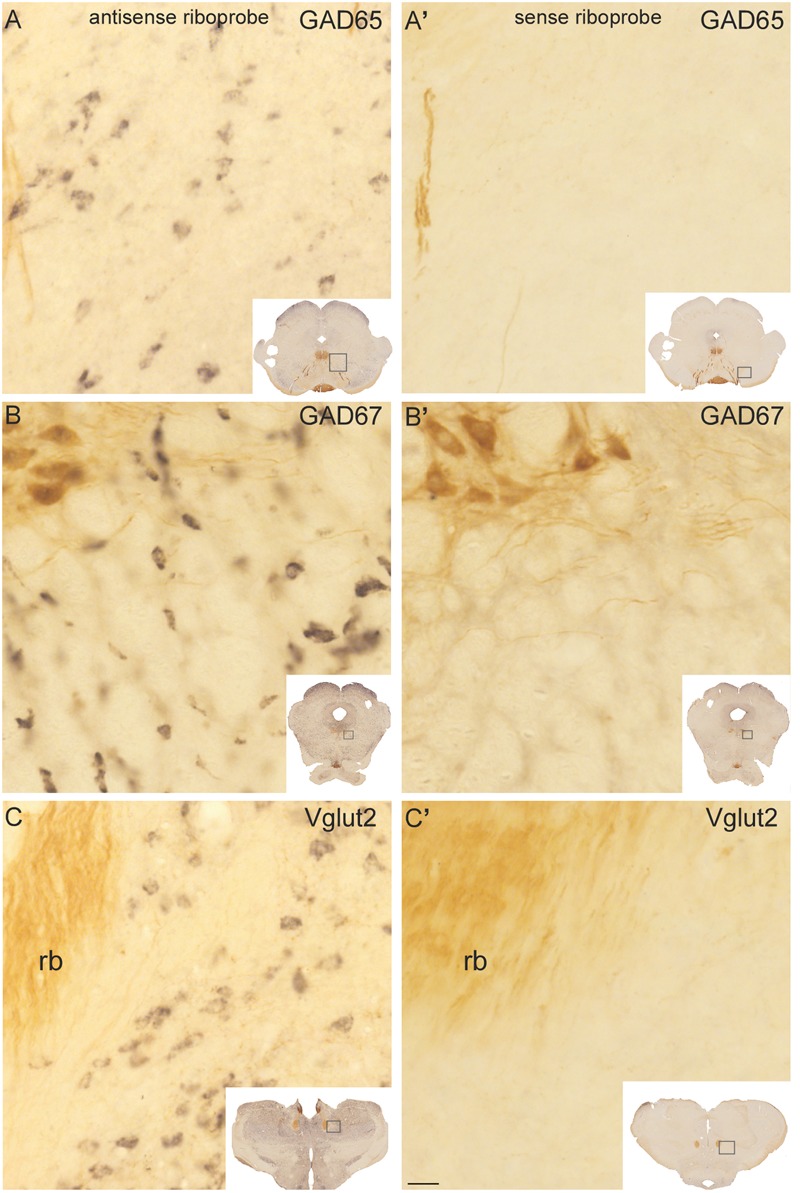
Control experiments for GAD65, GAD67, and Vglut2 riboprobes in sections processed dually for *in situ* hybridization and ChAT immunocytochemistry. Control sections hybridized with antisense **(A–C)** riboprobes against GAD65 **(A)**, GAD67 **(B)**, and Vglut2 **(C)** showed a specific black precipitate in neurons, whereas no reaction product was observed in the ones hybridized with any of the sense riboprobes **(A’–C’)**. ChAT-immunoreactive neurons and fibers (labeled in brown) were readily distinguishable in the trochlear nucleus (PPN; **B–B’**, upper left corner) and retroflex bundle (rb; **C–C’**, left), respectively. Insets: low magnification images of the control sections, showing within boxes the respective enlarged areas. Scale bar: 20 μm.

The stereological quantification was carried out in sections processed using a dual colorimetric protocol to visualize ISH for either GAD65, GAD67, or Vglut2 mRNA (Barroso-Chinea et al., 2007), followed by immunohistochemistry against ChAT. For ISH, the optimal concentrations of GAD65, GAD67, and Vglut2 sense and antisense riboprobes were first determined to ensure the specificity of the signal (**Figure 1**). Then, every one out of four sections containing PPN and/or LDT (14–15 sections per case) were selected and processed for the dual colorimetric protocol. Briefly, the free-floating sections were rinsed twice in 0.1 M PBS pH 7.4 with 0.1% active DEPC at RT. After pre-equilibrating in 5× SSC buffer (0.75 M NaCl and 0.085 M sodium citrate, pH 6.8), the sections were prehybridized at 58°C for 2 h in the hybridization solution [50% formamide (Sigma-Aldrich), 5× SSC, 40 μg/mL denatured salmon DNA, and 25% H_2_O-DEPC]. The biotinylated sense and antisense riboprobes were denatured for 8 min at 75°C, added to the hybridization solution at the following concentrations: 111 ng/ml (GAD65), 56 ng/ml (GAD67), or 222 ng/ml (Vglut2) and incubated at 58°C for 16 h. Following hybridization, the sections were rinsed thrice in 2× SSC at RT, 2× SSC at 65°C for 40 min, and 0.1× SSC at 65°C for 40 min and then immersed in a 94% methanol solution containing 0.4% H_2_O_2_ for 20 min at RT to remove endogenous peroxidase activity. The biotin-labeled probe was visualized using the standard TSA procedure (Bobrow and Moen, 2001; TSA^TM^ Biotin system, PerkinElmer, Boston, MA, United States). All incubations were carried out at RT, followed by rinses consisting of one rinse in TNT buffer (0.1 M Tris–HCl, pH 7.5, 0.15 M NaCl, 0.05% Tween 20) and two more in TN buffer (0.1 M Tris–HCl, pH 7.5, 0.15 M NaCl). The sections were equilibrated in TNB (0.5% blocking reagent in TN buffer) for 30 min and incubated in a solution containing streptavidin-conjugated HRP (1:100, TSA^TM^) in TNB buffer for 30 min. After rinsing, the sections were incubated for 10 min with biotinyl tyramide (1:50 in amplification diluent from TSA^TM^), rinsed again, and finally incubated with streptavidin-conjugated HRP (1:100) in TNB buffer for 30 min. After two rinses in TN buffer, they were equilibrated in Tris buffer (TB; 0.1 M Tris–HCl pH 7.6) for 5 min. The colorimetric detection of the biotin-labeled probe was achieved by a final incubation in TB containing (1) 0.024% of 3, 3′-DAB (Sigma), (2) 0.3% nickel ammonium sulfate, (3) 0.005% cobalt chloride, and (4) 0.0024% H_2_O_2_ for approximately 1 min, which yielded a fine granular black precipitate. The reaction was terminated by rinsing twice with TB. Subsequently, the sections were processed for ChAT immunoreactivity. Briefly, the sections were equilibrated in TS (0.1 M Trizma and 0.15 M NaCl, pH 7.6), preincubated for 1 h in a blocking solution containing 0.5% BSA in TS, and finally incubated overnight at RT in a solution containing goat anti-ChAT (polyclonal antiserum, AB-144P, Merck Millipore, Darmstadt, Germany; 1:500), 0.3% Triton X-100, and 0.1% BSA, in TS. After several rinses, the sections were incubated for 30 min in a 0.1% BSA solution in TS containing biotinylated donkey anti-goat IgG (1:250), rinsed again with TS, and incubated for 30 min in the avidin–biotin complex (ABC, Vector Elite Kit, Vector Laboratories, Burlingame, CA, United States). The bound peroxidase was developed with 0.022% DAB and 0.003% H_2_O_2_ in TB, yielding an amorphous brown precipitate. The reaction was stopped with TS, and after rinsing with PB, the sections were mounted and coverslipped using DPX.

### Dual Fluorescence Labeling of Single *in Situ* Hybridization and Immunocytochemistry

A dual fluorescence labeling protocol was used to investigate the potential colocalization of ChAT with any of the three riboprobes used. The protocol for the fluorescent detection of ISH was identical to that used for colorimetric detection except for the last incubation (90 min), in which streptavidin-HRP was substituted by Streptavidin-Alexa 488 (Molecular Probes Inc., Eugene, OR, United States; 1:100) in TNB buffer. The subsequent incubation in methanol/H_2_O_2_ was also carried out, as it enhances fluorescence labeling (Luquin et al., 2010). After several rinses with PBS, the sections were processed for immunofluorescence, undergoing first a preincubation in blocking solution containing 4% normal rabbit serum, 0.05% Triton X-100, and 4% of BSA for 40 min, followed by an overnight incubation in darkness at RT in the same solution containing anti-ChAT antibody (1:150). After three rinses with PBS, sections were incubated in darkness for 2 h in a solution containing 0.5% normal rabbit serum, 2% BSA, and an Alexa Fluor 568 rabbit anti-goat IgG (Invitrogen, ref: A-11079; 1:200). After three final rinses with PBS, the sections were mounted on gelatine-coated slides, air dried, defatted in toluene and coverslipped, and finally examined under a confocal microscope (LSM 510 META; Zeiss) using a 40× oil immersion lens with differential interference contrast.

### Triple Fluorescence Labeling of Dual *in Situ* Hybridization and Immunocytochemistry

A protocol of triple fluorescence labeling was used to establish the degree of colocalization of the two GAD isoforms and the potential coexpression of Vglut2 and GAD67 mRNAs within the cholinergic territory of PPN and LDT. Dual fluorescence ISH was performed with a mixture of (1) biotin-labeled antisense riboprobe for GAD65 and a digoxigenin-labeled probe for GAD67, or (2) biotin-labeled antisense riboprobe for GAD67 and a digoxigenin-labeled probe for Vglut2, or (3) biotin-labeled antisense riboprobe for Vglut2 and a digoxigenin-labeled probe for GAD67. The pre- and post-hybridization steps were identical to those described above for the colorimetric protocol except for the visualization of the probes. Alexa 633-conjugated streptavidin (Molecular Probes, 1:100) was first used to visualize the biotin-labeled riboprobe as previously described. Then the sections were briefly rinsed with TN buffer and incubated for 90 min at RT in darkness with an anti-digoxigenin antibody (1:500 in 0.5% blocking-TN; Roche Diagnostics GmbH, Mannheim, Germany) to visualize the digoxigenin-labeled riboprobe. After several rinses with detection buffer (100 mM Tris/HCl, pH 8, 100 mM NaCl, 10 mM MgCl_2_) in darkness at RT, the sections were incubated with HNPP/Fast Red TR substrate (Roche; peak emission at 562 nm) in detection buffer for 1 h. Finally, the cholinergic neurons were labeled as previously described, using Alexa Fluor 488 donkey anti-goat IgG (Molecular Probes, 1:200) as secondary antibody.

### Morphometry

The long and short axes of cells from each phenotype were measured at 100× in cells whose perimeter was clearly visible, using a specific tool available in NewCast software. This software enabled us to envision the orientation of the cell within the complete section and then mark the beginning and end of each axis when both points were in focus, while moving through the full depth of the section. This was facilitated by the fact that the reaction product labeling GAD65-, GAD67-, and Vglut2-positive cells tended to accumulate peripherally outlining the soma contour (Supplementary Figure S1). The minimum sample size (*n* = 50 neurons) was calculated using STATA (v. 12.0; StataCorp, College Station, TX, United States), accepting an alpha level of 5 and 80% power. Thus, 50 neurons from each subpopulation were measured in the PPN and another 50 in the LDT, selected from three sections from two cases (3 × 2).

### Stereological Cell Quantification

The optical fractionator method (West and Gundersen, 1990; Boyce et al., 2010) was used to obtain the total counts of the different cell subpopulations of PPN and LDT because it allows to establish cell numbers independently of V estimates, eliminating most potential biases due to tissue shrinkage. The first section for the neuronal counts was randomly selected from the first four containing the PPN, and then one every other four (160 μm) was systematically selected throughout the full rostrocaudal extent of PPN and LDT, resulting, on average, in 15 sections per animal. Once processed, the sections were analyzed using an Olympus Bx-UCB microscope (Olympus Optical Co, Europe GmbH, Hamburg, Germany) connected to a digital camera (DP71, Olympus) and supplied with a motorized microscope stage ProScan (Prior Scientific Inc., Rockland, MA, United States). The microscope was guided by a computer supplied with NewCast software (NewCast, v.2.16.1.0; Visioph+arm, Dermark) which provided a systematic, random, and uniform sampling of optical disectors across tissue sections. These were first examined at 2×; the closed contours of PPN and LDT were outlined at 10×, and cell counts were obtained using a 100× 1.4 NA oil-immersion objective.

The total cell counts of each neurochemical subpopulation were estimated using the following equation:

$$N\;=\;ssf\;\times \;asf\;\times \;hsf\;\times \;\Sigma Q^{-},$$

where *ssf* is the section sampling fraction, *asf* is the area sampling fraction, *hsf* is the height sampling fraction, and ΣQ^-^ denotes the cells counted in every region. *ssf* was calculated as *T*/BA, where *T* represents the distance between sections (160 μm) and BA is the block advance or thickness set at the microtome (40 μm; *asf* is calculated as (*D_x_* ×*D_y_*)/*a*, where *D_x_* and *D_y_* represent the step length in the *x* and *y* axes (155.49 μm in both cases), and *a* is the counting frame area (6,034 μm^2^); finally, *hsf* is calculated as *
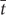
_Q_*^-^ /*h*, where *
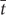
_Q_*^-^ is the number-weighted mean section thickness and *h* is the height of the disector. Cell number estimations obtained with the optical fractionator design are not affected by tissue shrinkage in the *X* and *Y* axes; however, to avoid a potential bias due to differential tissue deformation in the *Z* axis we used the number-weighted mean section thickness or *
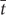
_Q_*^-^ (Dorph-Petersen et al., 2001; Bermejo et al., 2003). Finally, as the mean section thickness was 12.2 ± 1.7 μm disector height was set at 9 μm, keeping an upper guard zone of 2 μm and a lower one of variable height (1.2 μm on average).

Our counting unit was the equator plane of the cell soma, which is the plane of the cell with most sharp borders and it is normally visible in 1–2 microns thickness at most. A cell was counted if the equator was in focus within the height of the disector, which was automatically signaled by the program, and did not touch the forbidden sides (left and bottom) of the disector frame.

The sampling fraction was previously determined in a pilot study so as to ensure that a minimum of 100 cells per case from each neuronal phenotype were counted separately in the PPN and LDT, resulting in a coefficient of error (CE) ≤0.1 for each of them. The CE was calculated using equation 20 from Gundersen and cols (Gundersen et al., 1999). The number of disectors counted ranged between 3 in the smallest areas and 60 in the largest ones. The total number of cells counted in the five cases was 1,258 and 1,336 for ChAT-positive cells in PPN and LDT, respectively, 863 and 1,548 for GAD65-, 1,057 and 1,556 for GAD67-, and 1,260 and 1,134 for Vglut2-positive cells.

The V of PPN and LDT was calculated following the Cavalieri principle (Gundersen and Jensen, 1987) using the formula:

V = T × a × ΣP,

where *T* represents the distance between sections (160 μm), *a* is the area per point (0.024 mm^2^), and Σ*P* is the sum of points counted. Once the V was calculated, the cell densities (*N_v_*) were finally estimated using the formula: *N_v_* = *N*/*V*.

After the total cell counts were obtained, comparisons were carried out between the rostral and caudal portions of PPN and LDT in order to investigate potential regional differences in the distribution of the four cell subpopulations. To do this, we separately calculated in the rostral and caudal halves of both nuclei the mean number of cells per disector in all disectors previously counted, and compared the two means statistically. In series comprising an even number of sections, the first half of them were assigned to the rostral half and the second to the caudal one. In series comprising an odd number of sections, the extra one was systematically assigned to the rostral half of the nucleus.

### Statistical Analyses

All statistical analyses were carried out using SPSS (v. 15). Comparisons of the four subpopulations were made across cell types using either analysis of variance (ANOVA) and Tukey’s HSD *post hoc* test, or Kruskal Wallis and Mann–Whitney *U* tests with Bonferroni adjustment for *post hoc* paired comparisons, as applicable. For two group comparisons, either the *t*-test or Mann–Whitney *U* test was performed, as applicable.

### Quantification at the Confocal Microscope

In order to analyze the potential colocalization of Vglut2 and ChAT, every one out of six sections containing the PPN or LDT from five animals (∼10 and 3 sections per case, respectively) were selected and processed for a dual fluorescent protocol using a digoxigenin riboprobe for Vglut2 and immunofluorescence against ChAT. For the potential colocalization of GAD67 and Vglut2, six sections per case were selected from five animals and a triple fluorescent protocol was used combining two ISH and immunofluorescence against ChAT; here biotin was used for GAD67 and digoxigenin for Vglut2. Finally, the potential colocalization of GAD67 and GAD65 was analyzed in six sections per case from three animals. All images were taken under a confocal microscope (LSM 510 META; Zeiss) using a 40× oil immersion lens with differential interference contrast. The whole extent of both PPN and LDT was scanned and photographed, within the boundaries of the most peripheral cholinergic cells. A whole series of optical sections (Z-stack) was acquired from every field and the interval between every two slices was adjusted at 0.48 μm. Every Z-stack was analyzed slice by slice in order to investigate the potential colocalization of markers on the same plane. Single and dual labeled cells were counted using Zen lite 2012 software^1^.

## Results

### Specificity of Vglut2-, GAD65-, and GAD67-mRNA Labeling and ChAT Immunoreactivity

The specificity of the biotinylated riboprobes used for the detection of Vglut2, GAD65, and GAD67 transcripts was assessed in control experiments using the sense and antisense riboprobes. A cell-restricted black precipitate was observed in the sections incubated with the three antisense riboprobes (**Figures 1A–C**) but not in those incubated with the sense ones (**Figures 1A’–C’**), confirming the specificity of the riboprobes. Control sections were dually immunoreacted against ChAT using an antibody reported to colocalize with all ChAT mRNA-containing cells in the PPN and LDT (Wang and Morales, 2009), and in fact, the labeling of ChAT-positive cells and fibers exhibited the characteristic distribution of cholinergic cell groups and terminal fields in the diencephalon and brainstem (**Figures 1B,B’,C,C’**; Mesulam et al., 1983; Rye et al., 1987).

### Anatomical Delineation of the PPN and LDT

**FIGURE 2:**
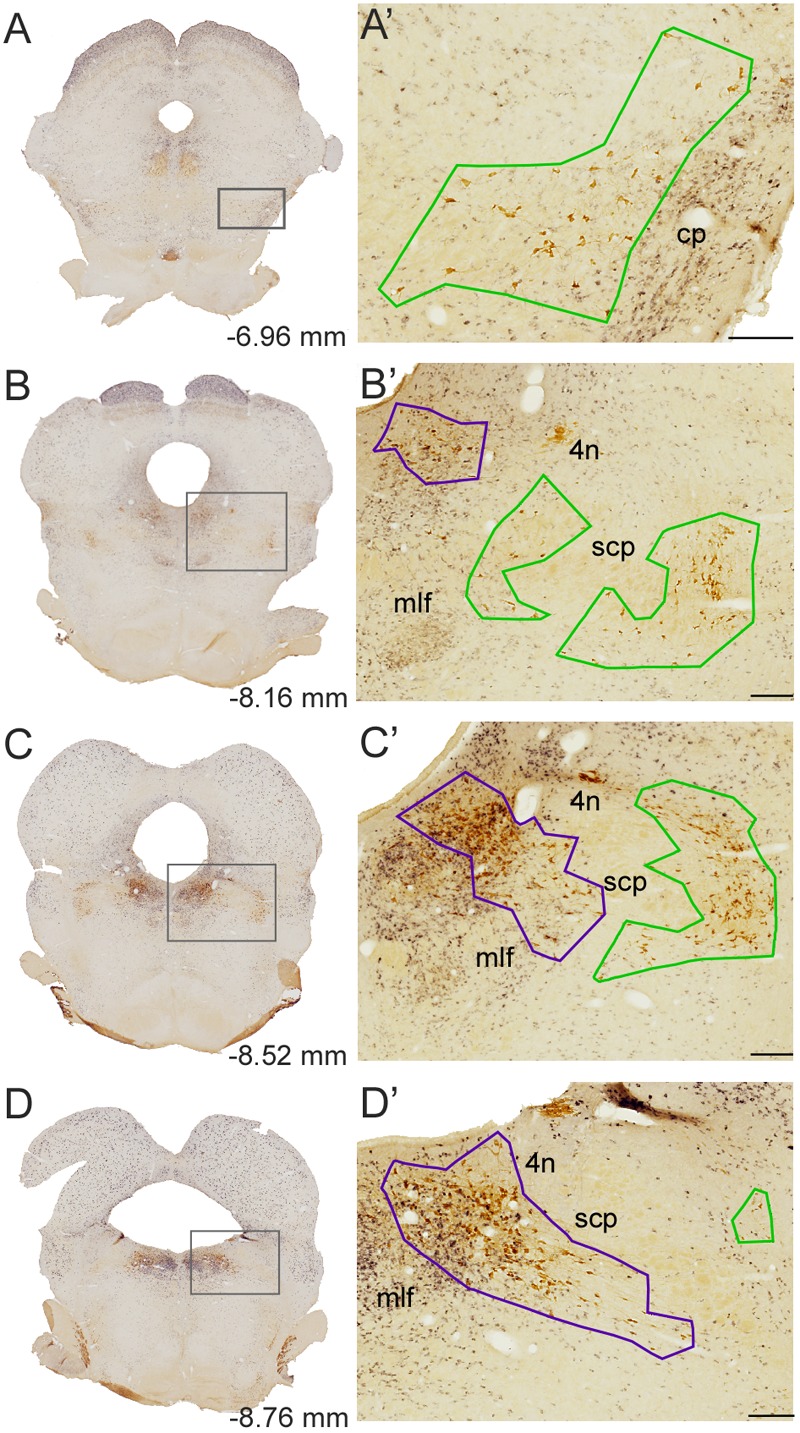
Delineation of the PPN and LDT. **(A–D)** Representative dually labeled sections in rostrocaudal sequence containing the PPN and LDT. Boxed areas are shown at higher magnification in **A’–D’**. **(A’–D’)** PPN (green) and LDT (purple) contours were drawn at 10×, prior to quantification. Delineations were carried out linking with straight lines the outermost cholinergic neurons. The PPN included the medial extension ventral to the scp (SPTg in Paxinos and Watson’s atlas; **B’,C’**) whereas the LDT also included LDTV **(C’,D’)**. scp, superior cerebellar peduncle; mlf, medial longitudinal fasciculus; 4n, trochlear nucleus. Scale bars: 200 μm. The scale bar in **D** corresponds to **B’–D’**.

The cytoarchitectonic boundaries of the PPN and LDT are difficult to determine, as the GABAergic and glutamatergic cells intermingled with the cholinergic neurons in both nuclei extend into the surrounding tegmentum without clear boundaries. Stereological analyses, however, require the precise delineation of the territories undergoing quantification. Thus, we have delineated the PPN and LDT outlining the area strictly contained within the most peripherally located ChAT-positive cells in each nucleus (**Figure 2**). The PPN was regarded as a single structure from its rostral pole in the ventral midbrain tegmentum (**Figures 2A,A’**) to its caudal pole in the pontine tegmentum, lateral to the scp, making no distinction between a *pars compacta* and a *pars dissipata* (**Figures 2C,C’,D,D’**). At mid-rostrocaudal levels, the PPN was traversed by the scp fibers; this intervening area was excluded from the territory delineated as PPN (**Figures 2B,B’**). The scattered cholinergic neurons ventral to the scp regarded as subpeduncular tegmental nucleus by Paxinos and Watson (SPTg; Paxinos and Watson, 2005) were considered here as an extension of PPN merging caudally with the LDT (Rye et al., 1987), and thus included within the territory of the former (**Figures 2B,B’,C,C’**). Regarding the LDT, both the LDT proper and the ventral LDT or LDTV (Paxinos and Watson, 2005) were considered part of a single LDT (**Figures 2C,C’,D,D’**).

### Morphology of the Different Cell Subpopulations in the PPN and LDT

Prior to the stereological quantification of the different cell subpopulations in the PPN and LDT, we carried out a morphometrical analysis of the diverse cell groups, which were identified by the expression of ChAT, GAD65, GAD67, or Vglut2. The long and short axes of neurons (*n* = 50) from each cell subpopulation were measured and their respective mean values compared between PPN and LDT, and also across phenotypes (**Table 1**). In relation to previously reported morphometrical data, the mean value of the long axis of the cholinergic neurons obtained here (**Table 1**) was similar to most of the reported values (Spann and Grofova, 1992; Ford et al., 1995; Boucetta and Jones, 2009) and slightly higher than those described by Rye et al. (1987).

**Table 1:** Morphometric analysis of PPN and LDT subpopulations^∗^.

	PPN		LDT	
	Long axis	Short axis	Long axis	Short axis
ChAT+	22.5 ± 3.8^¶^	12.17 ± 2.^¶ §^	21.9 ± 3.2**^†^**	13.95 ± 2.3^†§^
GAD65+	15.3 ± 2.5	9.6 ± 1.4^§^	15.8 ± 2.3	10.7 ± 1.4^§^
GAD67+	15.9 ± 2.6	9.4 ± 1.5	15.9 ± 2.2	9.3 ± 1.2
Vglut2+	15.3 ± 0.3	8.9 ± 1.3^§^	15.8 ± 0.3	9.8 ± 1.4^§^

*^∗^Mean ± SD obtained from a total of 50 neurons from two different cases.*

*^¶^ Significantly higher than the same axis in the other three subpopulations within the same nucleus (ANOVA, *p* < 0.01 followed by Tukey’s test). **^†^**Significantly higher than the same axis in the other three subpopulations within the same nucleus (ANOVA, *p* < 0.01 followed by Tukey’s test). ^§^Significant differences are found in this parameter between the PPN and LDT (Student’s *t*-test, *p* < 0.01). All other comparisons were not statistically significant.*

A comparison between the two cholinergic subpopulations, however, revealed that the short axis was significantly smaller in the PPN cells than in the LDT ones (*p* = 0.000; **Table 1**). These data extend previous qualitative observations describing cholinergic cells in PPN as elongated or fusiform, in comparison to the more radially symmetrical cells in LDT (Mesulam et al., 1983).

The comparative analysis between cholinergic and non-cholinergic cells showed that both long and short axes of ChAT-positive neurons were significantly higher than those of Vglut2-, GAD65-, and GAD67-positive cells in the PPN as well as in the LDT (**Table 1** and Supplementary Figures S2, S3).

Regarding the non-cholinergic neurons, GAD65- and GAD67-positive cells were small-to medium-sized (**Table 1**) consistent with previous data (Ford et al., 1995; Boucetta and Jones, 2009) and so were Vglut2-positive neurons (**Table 1**); in fact, no size differences were found among these cells either within the PPN or LDT (**Table 1** and Supplementary Figures S2, S3). However, when single subpopulations were compared between the two nuclei, both GAD65- and Vglut2-positive neurons in the PPN showed significantly smaller values for the short axis than in LDT (**Table 1**), indicating morphological differences between these cells in the two nuclei.

### Stereological Estimations of Total Cell Numbers of Each Phenotype in the PPN and LDT

**FIGURE 3:**
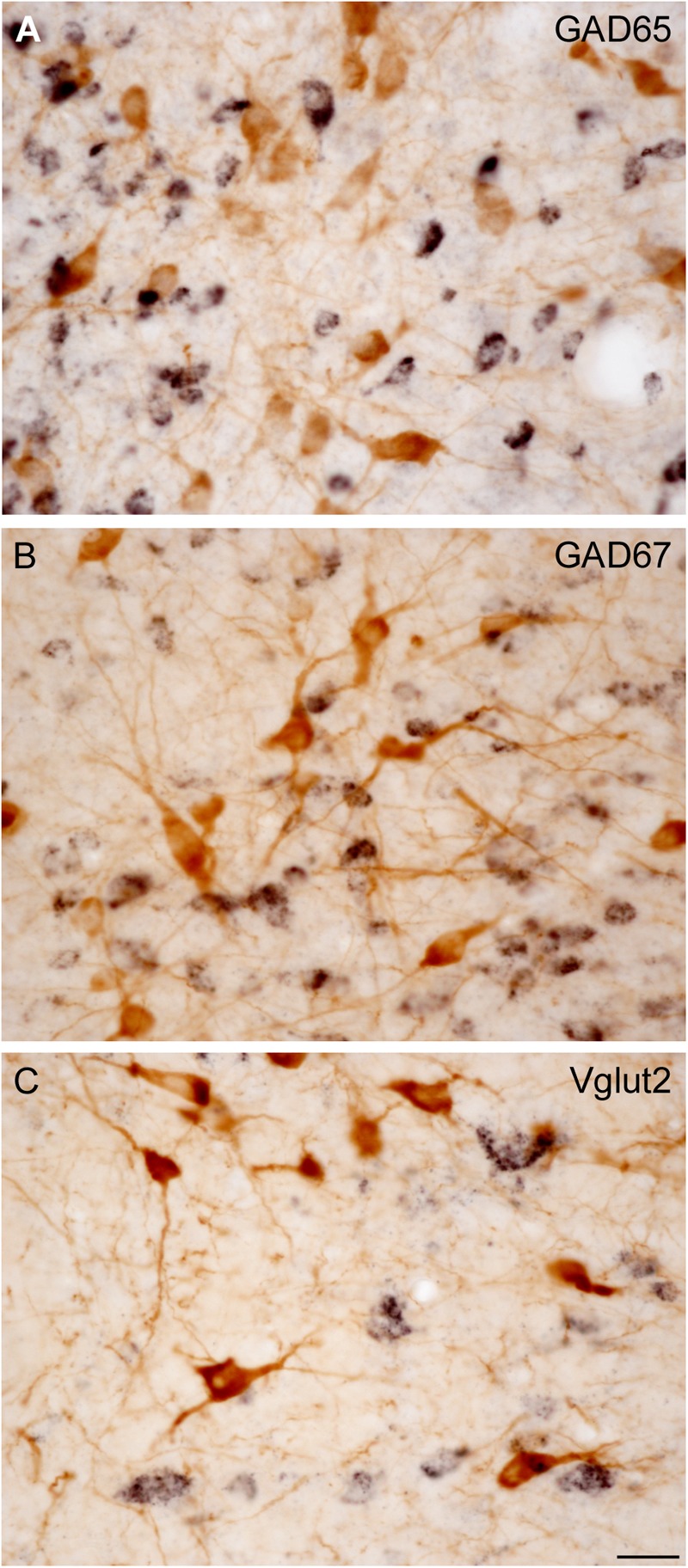
Direct visualization of ChAT-immunoreactive cells and GAD65-, GAD67-, or Vglut2-positive cells in the PPN and LDT. **(A–C)** Microphotographs show examples of dually labeled sections used for quantification. This protocol allowed to directly and distinctly visualize both ChAT-immunoreactive neurons (**A–C**, brown cells) and biotin-labeled GAD65- **(A)**, GAD67- **(B)**, or Vglut2-positive neurons **(C)** displaying a black granular labeling. Scale bar: 20 μm.

**FIGURE 4:**
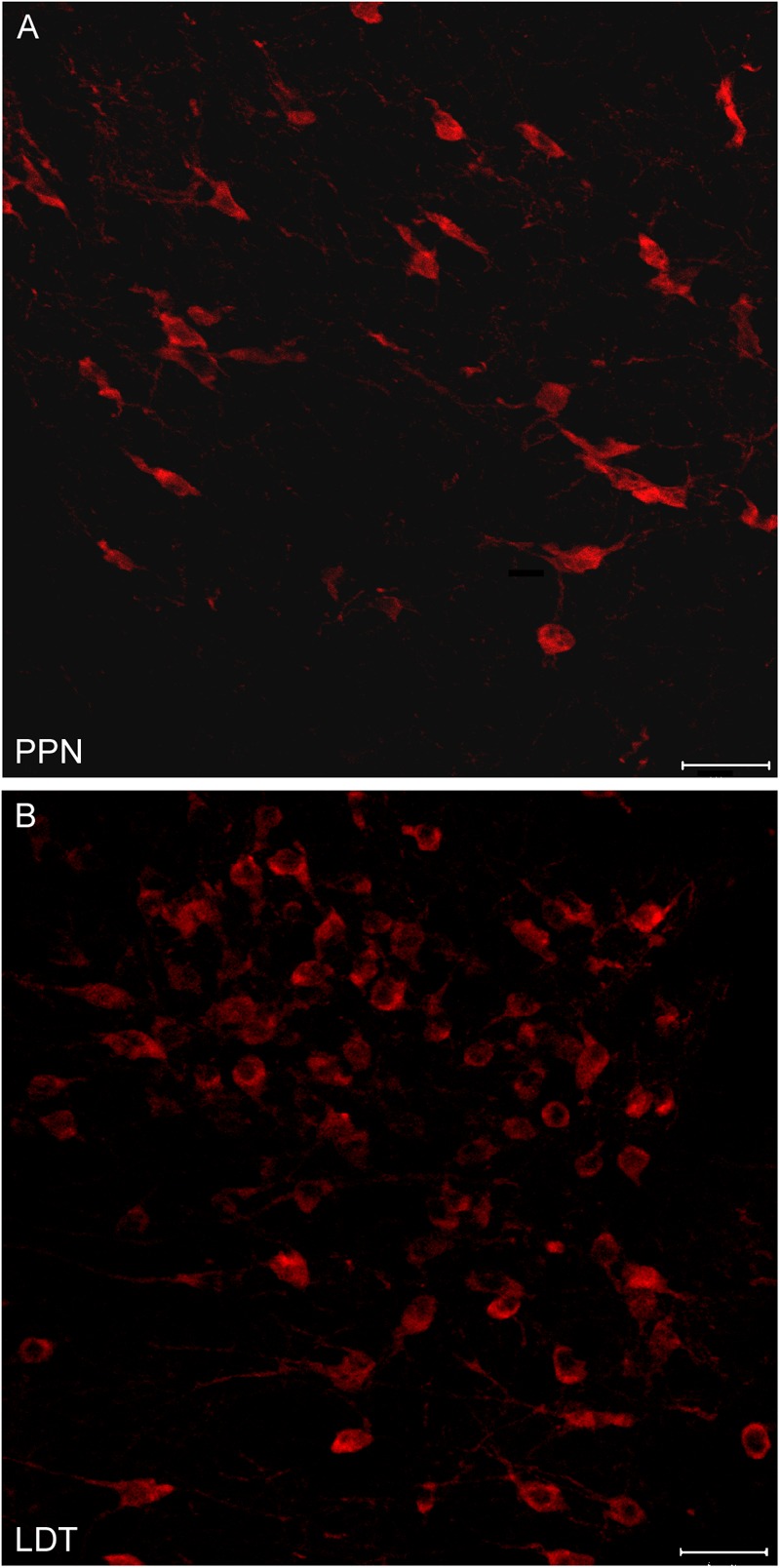
Different neuronal density of cholinergic cells in the PPN and LDT. **(A,B)** Confocal z-stack images of ChAT-immunoreactive neurons from the **(A)** PPN and **(B)** LDT. Neurons in the PPN follow the orientation of the superior cerebellar peduncle fibers, and are generally less densely packed than cells in the LDT. Scale bar: 20 μm.

All stereological counts were carried out on dually labeled sections, where the brown ChAT-positive cells were readily distinguishable from those containing the black granular reaction product labeling either GAD65 (**Figure 3A**), GAD67 (**Figure 3B**), or Vglut2 (**Figure 3C**) mRNA. Both DAB-brown and DAB-nickel reaction products were present across the full thickness of the section, in all sections counted. The direct and distinct visualization of both cholinergic and non-cholinergic cells in single sections facilitated their unambiguous identification, enabling the delineation of PPN and LDT territories first, and the subsequent reliable count of cholinergic and non-cholinergic cells. The total number of cholinergic neurons in the PPN and in the LDT was similar (**Table 2**), although the cell density of ChAT-positive cells in LDT was almost twice that of the PPN (**Table 2**). These data confirm and extend our qualitative observations (**Figure 4**) as well as those of others (Mesulam et al., 1983; Rye et al., 1987), indicating a higher cell density of cholinergic cells in the LDT than in the PPN.

**Table 2:** Estimated number, volume, and density of cholinergic cells in the PPN and LDT.

Mean estimations (*n* = 8)	PPN^∗^	LDT^∗^
Number of cells	3,360 ± 590	3,650 ± 976
Volume (mm^3^)	0.65 ± 0.12	0.35 ± 0.09
Density (neurons × 10^3^/mm^3^)	5.19 ± 0.71	10.60 ± 1.53

*^∗^Mean ± SD. Data were obtained from eight animals.*

**FIGURE 5:**
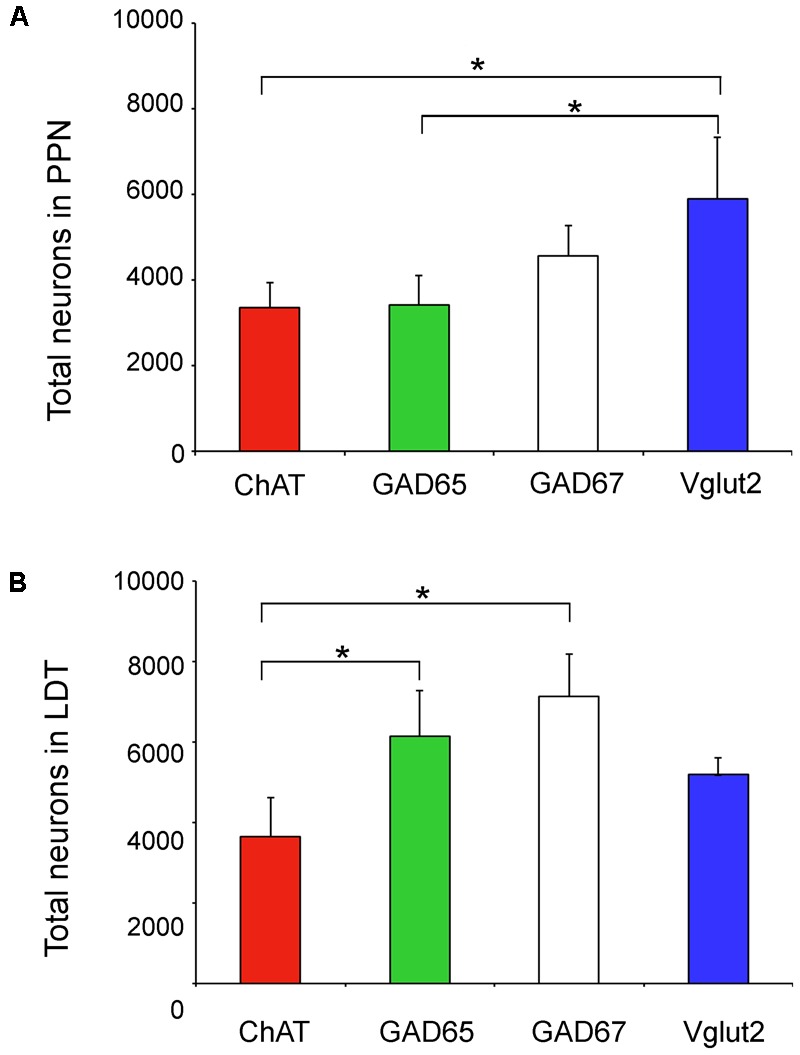
Total number of neurons of the four phenotypes analyzed in the PPN and LDT. **(A)** Graph showing the mean total number of neurons in the PPN. The estimated number of Vglut2-positive cells was significantly higher than that of GAD65- and ChAT-positive subpopulations (Kruskal Wallis, *p* = 0.0003). **(B)** Parallel data in the LDT; here the estimated total number of ChAT-positive neurons was significantly lower than that of GAD65 and GAD67 subpopulations (Kruskal Wallis, *p* = 0.0004). Asterisks indicate statistically differences among groups.

The analysis of non-cholinergic cells showed that the total number of GAD65-positive neurons in the PPN was similar to that of the cholinergic population, while GAD67-positive cells outnumbered the latter by 1.4 times (**Table 3** and **Figure 5A**). However, Vglut2-positive cells were the most abundant cell phenotype in the PPN (**Table 3** and **Figure 5A**); in fact, the estimated number of Vglut2-positive cells was significantly higher than that of GAD65- and ChAT-positive subpopulations (Kruskal Wallis, *p* = 0.0003). In the LDT, the number of Vglut2-positive cells was also higher than that of cholinergic cells by 1.4 times (**Table 3** and **Figure 5B**), but interestingly, GAD65-positive cells almost doubled the cholinergic subpopulation (**Figure 5B**), and so did GAD67-positive cells, indicating that GAD-expressing and thus GABAergic cells are the predominant cell phenotype in the LDT. These data reveal that the proportion of the three cell phenotypes in the PPN is different from that in the LDT.

**Table 3:** Estimated number of neurons and neuronal densities of the different cell phenotypes in the PPN and LDT.

	PPN			LDT		
Neuronal phenotype	Total *n* of neurons	Density (×10^3^/mm^3^)	CE	Total *n* of neurons	Density (×10^3^/mm^3^)	CE
ChAT+ (*n* = 8)	3,360 ± 590	5.19 ± 0.7	0.083	3,650 ± 976	10.60 ± 1.5	0.082
GAD65+ (*n* = 5)	3,420 ± 688	5.20 ± 0.8	0.079	6,140 ± 1,136	17.3 ± 1.9	0.059
GAD67+ (*n* = 5)	4,570 ± 710	6.44 ± 1.3	0.085	7,140 ± 1,059	20.07 ± 4.1	0.059
Vglut2+ (*n* = 5)	5,910 ± 1,425	9.72 ± 1.6	0.067	5,190 ± 420	16.83 ± 4.02	0.068

*Data are expressed as mean ± SD; *n*, number of cases analyzed; CE, mean coefficient of error.*

### Coexpression of Cholinergic and Non-cholinergic Markers in the PPN and LDT

**FIGURE 6:**
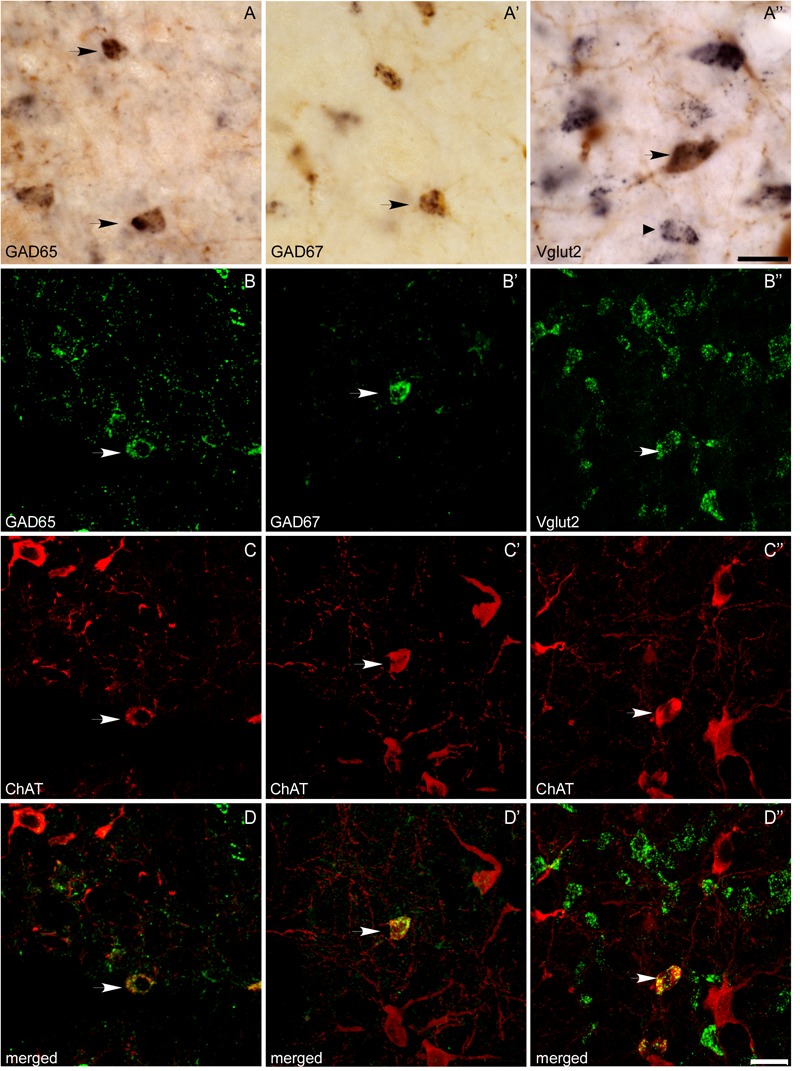
Colocalization of ChAT immunoreactivity and GAD65, GAD67, or Vglut2 mRNA expression in the PPN and LDT. **(A–A”)** Microphotographs of colorimetric dual labeling taken at the magnification used for quantification (100×). Arrows mark potential dually labeled cells recorded during the stereological quantification, while a small arrow points to a single Vglut2-positive cell. Scale bar: 20 μm. **(B–D,B’–D’,B”–D”)** Confocal images of immunofluorescence labeling, confirming the colocalization of ChAT immunoreactivity with either GAD65 **(B–D)**, GAD67 **(B’–D’)**, or Vglut2 **(B”–D”)** mRNAs, although the degree of colocalization was low. Scale bars: 20 μm.

In addition to the single labeled neurons observed in dually labeled sections, cells showing black granules of ISH reaction product within brown-filled ChAT-positive neurons were also observed at 100× (**Figures 6A,A”**). As the number of these potentially double labeled cells was too low to be quantified using stereology, we counted those located within the counting frames previously used to count the single labeled ones, and calculated the corresponding percentage of dually versus single labeled cells per case, and the mean percentage in the five cases (**Table 4**). Our results indicated that 2 and 3% of ChAT-positive cells in the PPN coexpressed GAD65 and GAD67, respectively, while 7.5% of ChAT-positive neurons coexpressed Vglut2 (**Table 4**). Similarly, ∼3% of ChAT-positive neurons in the LDT coexpressed either GAD65 or GAD67, while 5% of them coexpressed Vglut2 (**Table 5**). The coexpression of markers for each of the three mRNA transcripts and ChAT-immunoreactivity was confirmed by confocal microscopy (**Figures 6B–D,B’–D’,B”–D”**). As the potential coexpression of ChAT and Vglut2 ranged between 5 and 10%, we performed a semiquantitative analysis of dually labeled cells in confocal images, to reliably establish the degree of colocalization in both nuclei. The analysis of 150–200 neurons per case (*n* = 5) revealed that 7.7% of ChAT- positive neurons in the PPN and 5.3% in the LDT colocalized Vglut2 (**Table 6**), confirming the previous estimations obtained using colorimetric methods.

**Table 4:** Total numbers and percentages of dual ChAT^+^/mRNA^+^ neurons in the PPN.

mRNA	mRNA^+^ neurons (*n*)	ChAT^+^ neurons (*n*)	Dually labeled neurons (*n*)	Percentage of ChAT^+^ neurons coexpressing mRNA
GAD65+	172.6 ± 44.4	174.4 ± 36.9	3.2 ± 1.6	1.96% ± 1.3
GAD67+	196.4 ± 47.7	151.6 ± 51.5	4 ± 2.2	2.91% ± 1.8
Vglut2+	232 ± 98.3	125.8 ± 26.3	10.2 ± 3.2	7.48% ± 1.8

*Potential dually labeled cells were observed and counted during stereological quantification of single labeled ones in the PPN. As their low numbers precluded stereological estimations, we have calculated the relative percentages of these cells comparing directly the “within frame” counts of dual labeled cells with the concurrent “within frame” counts of single labeled ones. Data are expressed as mean ± SD, and were obtained from five animals.*

**Table 5:** Total numbers and percentages of dual ChAT^+^/mRNA^+^ neurons in the LDT.

mRNA	mRNA^+^ neurons (*n*)	ChAT^+^ neurons (*n*)	Dually labeled neurons (*n*)	% of ChAT^+^ neurons coexpressing mRNA
GAD65	301.2 ± 40.2	179.5 ± 13.9	5 ± 2.8	2.68% ± 1.3
GAD67	311.2 ± 1	157.6 ± 34.2	4.2 ± 2.4	2.46% ± 1
Vglut2	240.8 ± 34.4	132.2 ± 39.4	7.6 ± 1.8	5.1% ± 1.5

*Potential dually labeled cells were observed and counted during stereological quantification of single labeled ones in the LDT. As their low numbers precluded stereological estimations, we have calculated the relative percentages of these cells comparing directly the “within frame” counts of dual labeled cells with the concurrent “within frame” counts of single labeled ones. Data are expressed as mean ± SD, and were obtained from five animals.*

**Table 6:** Total counts of ChAT-positive and dual ChAT^+^/Vglut2^+^ neurons in the PPN and LDT obtained using fluorescence labeling.

	PPN			LDT		
Case	ChAT^+^	ChAT^+^/Vglut2^+^	%	ChAT^+^	ChAT^+^/Vglut2^+^	%
R370	181	14	7.74	164	9	5.48
R371	205	16	7.80	109	6	5.50
R389	227	18	7.93	171	8	4.68
R390	194	15	7.73	155	9	5.81
R391	200	15	7.5	164	8	4.88
**Mean**	**201.4**	**15.6**	**7.74**	**152.6**	**8**	**5.27**

### Coexpression of GAD65 and GAD67 mRNAs in the Majority of PPN and LDT GABAergic Neurons

**FIGURE 7:**
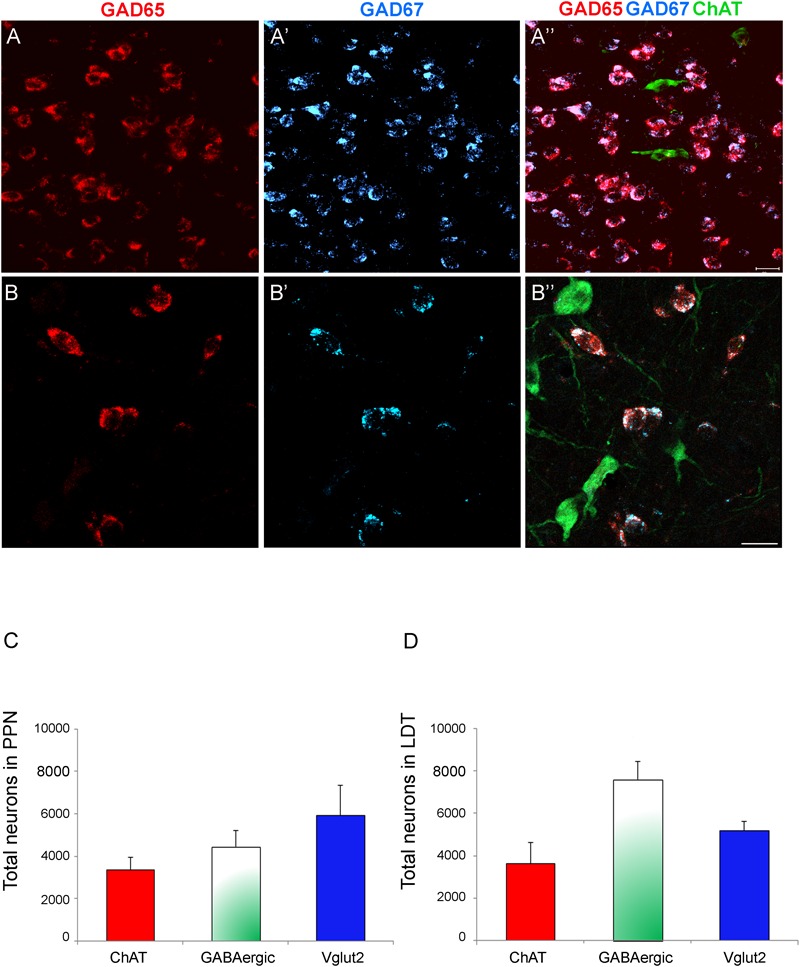
High degree of colocalization of GAD65 and GAD67 in PPN and LDT neurons. **(A–B”)** Confocal images of triple labeling in the PPN showing GAD65 expression only (**A,B**, red channel),, GAD67 expression only (**A’,B’**, blue channel), and the merged image of the three channels (**A”,B”**, cholinergic cells in green). The merged image shows the high degree coexpression of GAD67 and GAD65 mRNAs in GABAergic cells (pink neurons). Scale bar: 20 μm. **(C–D)** Graphs showing the relative densities of the three cell phenotypes in the PPN **(C)** and LDT **(D)**. The estimation of the total GABAergic cell density was calculated from the data of single GAD65 and GAD67 neuronal densities and the degree of colocalization of the two GAD isoforms.

Next we investigated the extent of colocalization of GAD65 and GAD67 mRNA expression in the PPN and LDT, in order to determine the total number of GABAergic cells in both nuclei. The semiquantitative analysis showed that 87.3% of neurons (696 out of 797) in the PPN coexpressed GAD65 and GAD67 mRNAs, 9.1% were exclusively GAD67-positive, and 3.6% expressed only GAD65 mRNA. Similarly, in the LDT, 85.5% of neurons (714 out of 835) coexpressed both GAD isoforms, 10.8% only GAD67, and 3.7% only GAD65. Thus, a vast majority of cells coexpressed the two markers, while 13–15% of the GABAergic cell subpopulation expressed only one of the two GABA synthesizing enzymes, predominantly GAD67 (**Figures 7A–A”,B–B”**). With these data, the total number of GABAergic cells in the PPN and LDT was calculated (*n* = 4,440 ± 699 and 7,600 ± 1,097, respectively) and plotted in relation to that of cholinergic and glutamatergic cells in the two nuclei (**Figures 7C,D**).

### Lack of Coexpression of GAD67 and Vglut2 mRNAs in the PPN and LDT

**FIGURE 8:**
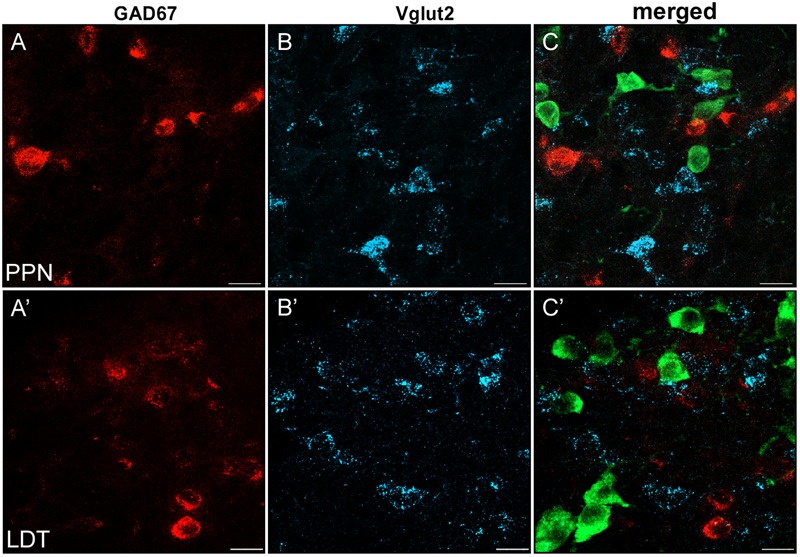
Virtual lack of colocalization of GAD67 and Vglut2 in PPN and LDT. **(A–C)** Single plane confocal images from the PPN showing GAD67 (**A**, red channel), Vglut2 (**B**, blue channel), and the triple labeled merged image **(C)**. **(A’–C’)** Confocal images of immunofluorescence labeling in LDT. **(C–C’)** Merged images of single planes showing the lack of colocalization of GAD67 (red) and Vglut2 mRNAs (blue). ChAT-positive neurons (green) helped to localize the anatomical boundaries of both PPN and LDT. Scale bar: 20 μm.

Specific cell subsets of the rat entopeduncular nucleus and ventral tegmental area coexpress either GAD65 or GAD67 and Vglut2 (Barroso-Chinea et al., 2008; Root et al., 2014), and seem to functionally corelease both glutamate and GABA in some of their target areas (Root et al., 2014; Shabel et al., 2014; Ntamati and Lüscher, 2016). To determine whether GABAergic and glutamatergic markers were also colocalized in the PPN or LDT, we carried out a triple labeling experiment and the subsequent quantification (**Figure 8**). As 95% of the total GABAergic cells in both nuclei are GAD67-positive, the GAD67 riboprobe was used for the analysis of the potential colocalization. A total of 1,270 GAD67- and 475 Vglut2-positive neurons were analyzed in the PPN, and 579 and 147 in the LDT, respectively. None of the cells analyzed showed coexpression of markers, suggesting that GAD67-positive and Vglut2-positive subpopulations in the two tegmental nuclei are independent (**Figure 8**).

### Cell Composition of the PPN and LDT

**FIGURE 9:**
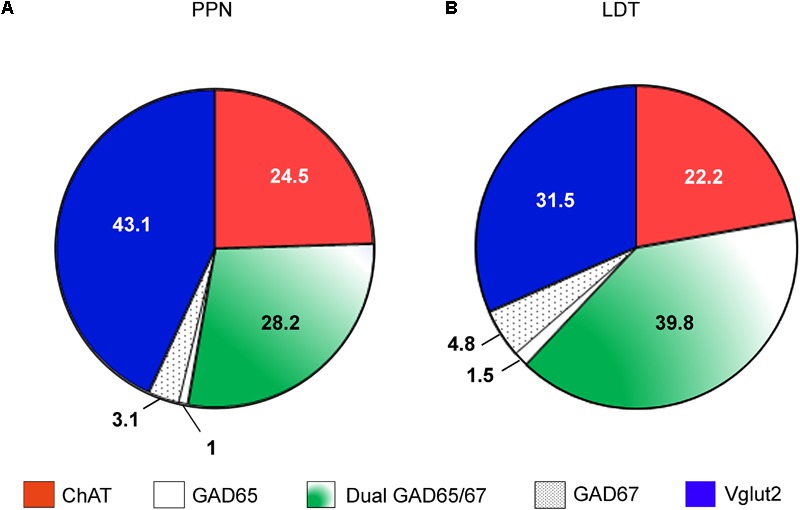
Estimated cell composition of the PPN and LDT. Percentual estimations of the cell composition of both the PPN **(A)** and LDT **(B)** are presented as a summary of the data obtained in the present study. The largest subpopulation in the PPN is glutamatergic, while in the LDT, it is GABAergic.

From the stereological estimates of the total cholinergic, glutamatergic, and GABAergic neurons in the PPN and LDT, we calculated the cell composition of each nucleus (**Figures 9A,B**). The cholinergic neurons comprised the smallest population in both the PPN and LDT, representing 24.5 and 22.2% of the total size, respectively; the glutamatergic population was the largest one in the PPN, accounting for 43.1% of the total cells (**Figure 9A**), while the GABAergic neurons were the most abundant phenotype in the LDT (46.2% of the total; **Figure 9B**).

### Topographical Distribution of the PPN and LDT Cell Subpopulations

PPN cholinergic neurons are preferentially located in the caudal portion of the nucleus, contrarily to the GABAergic cells (Mena-Segovia et al., 2009). To determine whether this was also the case in our material and extend the analysis to the glutamatergic subpopulation, we calculated the mean number of cells/disector in all disectors previously counted in the rostral half of the nucleus, and then in those in the caudal half of the nucleus in both PPN and LDT, and compared the two means statistically. In agreement with the previous report (Mena-Segovia et al., 2009), we confirmed the significantly higher number of ChAT-positive neurons per disector in the caudal portion of the PPN (Wilcoxon signed-rank test *p* < 0.05), as well as a preferential rostral distribution of GABAergic cells. Specifically, we found that both GAD65-positive neurons and GAD67-positive neurons displayed a significantly higher number of cells in the rostral PPN (Wilcoxon signed-rank test *p* < 0.05 in both groups). Finally, the comparison of the rostral versus caudal distribution of VGlut2 positive cells revealed that the mean density in the caudal half of PPN was significantly higher than that in the rostral one (Wilcoxon signed-rank test for paired samples, *p* < 0.05). These data demonstrate that the three cell phenotypes in the PPN display a topographical distribution throughout the rostrocaudal axis.

In the LDT, neither the cholinergic nor the glutamatergic subpopulations showed a preferential regional distribution, while both GAD65-positive and GAD67-positive cell groups were preferentially located in the rostral half of the nucleus (Wilcoxon signed-rank test *p* < 0.05).

## Discussion

The estimates of total cell numbers in the PPN and LDT obtained here reveal structural similarities between these two functionally associated brainstem nuclei: both have virtually the same total number of cholinergic neurons and, in both cases, this subpopulation is actually the smallest of the three analyzed here. In addition, the two nuclei show a parallel distribution of GAD isoforms among their GABAergic subpopulations, wherein >85% of cells coexpress GAD65 and GAD67, ∼10% expresses only GAD67, and <5% expresses only GAD65. Finally, a small but significant amount of cholinergic neurons in both nuclei coexpresses Vglut2 in a similar proportion. Together with this, the PPN and LDT show clearly distinct neurochemical cell compositions: the most abundant cell phenotype in the PPN is glutamatergic, while GABAergic cells comprise the largest cell phenotype within LDT. This difference in their relative cell phenotypes likely contributes to some of the functional differences reported between the two nuclei. Besides, we also report for the first time that the total glutamatergic subpopulation – similarly to the cholinergic one – shows a significant preferential distribution in the caudal PPN, emphasizing the existence of functional territories within this nucleus.

### Total Counts in PPN and LDT and Differences in the Neurochemical Compositions of the Two Nuclei

The present study (1) provides the first stereological estimations of the total number of glutamatergic cells in the PPN, (2) confirms previous stereological cell counts on PPN cholinergic cells, and (3) extends previous estimations of GABAergic cells in this nucleus by providing separate estimations of GAD67 and GAD65 cell counts.

The stereological estimation of the total number of glutamatergic cells in the PPN is important because it completes the stereological counts of the three main PPN subpopulations, enabling us to make an estimation of the relative percentages of each of three cell phenotypes in this nucleus; interestingly, this calculation revealed that the glutamatergic phenotype was the most abundant one in the PPN. The relative percentages of the three phenotypes – glutamatergic, cholinergic, and GABAergic – obtained here (43, 25, and 32) were almost identical to those reported in a previous study which did not use stereological methods (43, 27, and 31; Wang and Morales, 2009). Thus, the present study confirms with unbiased methods that the PPN cholinergic subpopulation is the smallest of the three cell phenotypes and the glutamatergic one, the largest, in agreement with the previous report (Wang and Morales, 2009).

As recently shown in an elegant study, the glutamatergic subpopulation of PPN is the main recipient of monosynaptic inputs from diverse basal ganglia nuclei (Roseberry et al., 2016). In turn, this subpopulation is the origin of a large number of projections to the STh and basal forebrain (Kita and Kita, 2011; Martinez-Gonzalez et al., 2014; Kroeger et al., 2017). Actually, PPN projections to the STh have been reported to be nine times more abundant from non-cholinergic – likely glutamatergic – neurons than from cholinergic ones (Bevan and Bolam, 1995; Kita and Kita, 2011). The fact that glutamatergic cells almost double the number of cholinergic cells in the PPN provides an anatomical substrate in support of the reported neurochemical phenotype of these projections.

Regarding previous cell counts of cholinergic and GABAergic cells in the PPN, our estimation of the total number of cholinergic neurons (3,360 ± 590) is slightly higher but consistent with the one obtained in two previous stereological studies (*n* = 2,942 ± 122; Mena-Segovia et al., 2009; 2,907 ± 112, Pienaar and van de Berg, 2013). In contrast, our estimation of the total GABAergic cells calculated from the neuronal densities of GAD65- and GAD67-positive cells and their degree of colocalization accounts for ∼2/3 of the one obtained in the only previous report (4,440 ± 699 neurons here versus 6,571 ± 818 in Mena-Segovia et al., 2009). This divergence is likely due to differences in the nuclear delineation of PPN between the two studies. Thus, Mena-Segovia et al. (2009) demarcated the territory of PPN at a distance of 50 μm from the cholinergic neurons while our delineation was carried out strictly around them and without smoothening the contour; in addition, their delineation seems to comprise the territory occupied by the scp in the different sagittal sections, while here it was excluded of PPN boundaries despite the fact that the scp area, in addition to fiber bundles, also contains intermingled cells. These differences suggest that the territory regarded as PPN by Mena-Segovia et al. (2009) is larger than ours, and this would specifically affect the number of non-cholinergic cells. The aim of the present work was to obtain unbiased cell counts of the PPN and LDT as a basis for future studies; therefore, we confined the quantification to a territory that could be easily and consistently delineated in subsequent studies, enabling to establish reliable correlations. For this reason, we did not include in the area of quantification either, the area that lies medial to the territory demarcated by the most peripheral cholinergic cells of the PPN. Although this medial area is anatomically and functionally an integral part of the nucleus, both as recipient of nigral and pallidal efferents, and as source of PPN projections to the STh (Hall et al., 1989; Spann and Grofova, 1991; Grofová and Zhou, 1998; Pérez-Lorenzo and Mengual, 2006; Kita and Kita, 2011), it largely contains glutamatergic and GABAergic cells that are continuous with those in the surrounding tegmentum; therefore, its boundaries could not be clearly established using either cytoarchitectonic or neurochemical criteria.

With regard to the LDT, the present results provide the first stereological estimations of cholinergic, glutamatergic, and GABAergic cells in this nucleus. Based on these data, we have also estimated the relative percentages of each neurochemical subgroup. The relative percentages obtained here (32, 22, and 46 for glutamatergic, cholinergic, and GABAergic cells, respectively) reveal that, in marked contrast to the PPN, the GABAergic cells are the most abundant ones in the LDT, accounting for ∼50% of LDT cells. These data are similar to those obtained in a previous quantitative study, although the relative percentage of GABAergic cells was lower (Wang and Morales, 2009).

Few studies have investigated the phenotype of non-cholinergic projections arising from the PPN or LDT, partly due to the difficulty in identifying either the glutamatergic or GABAergic cells. One of them, however, reported that ∼11% of PPN and LDT cells projecting to the lateral hypothalamus were GAD-ir, percentage that raised to 23% if only the LDT was considered (Ford et al., 1995). Such an increase in GABAergic output when the LDT alone was compared with PPN and LDT together would be consistent with the present data, given the higher percentage of GABAergic cells in the LDT with respect to the PPN.

Functional and behavioral differences have been reported between the PPN and LDT (Norton et al., 2011; Redila et al., 2015; Dautan et al., 2016; Xiao et al., 2016). Some of them can be accounted for by the fact that some cholinergic efferents from each nucleus end on different target structures as well as on different subsets of neurons within the same nucleus, and participate in segregated circuits (Satoh and Fibiger, 1986; Hallanger et al., 1987; Hallanger and Wainer, 1988; Dautan et al., 2016; Xiao et al., 2016). In addition, the percentage of cholinergic cells projecting to the same nucleus may vary, like in the ventral substantia nigra compacta, where it is significantly higher in the PPN than in LDT (Xiao et al., 2016). The disparity between the relative percentages of the three cell phenotypes in PPN and LDT reported here represents a remarkable anatomical difference between the two nuclei that adds to the connectional and topographical dissimilarities just mentioned, likely contributing to their functional diversity. Additional anatomical observations recently reported, like the higher number of cells in PPN – both cholinergic and non-cholinergic – expressing the GABA_A_ receptor α1 subunit (Luquin et al., 2015), as well as the higher number of non-cholinergic cells in the PPN, both glutamatergic and GABAergic, expressing the γ2 subunit (Paternain et al., 2017) in comparison to the LDT, may also contribute to clarify the internal organization of the two nuclei at the cellular level and to better understand their specific functions.

### Functional Territories Within the PPN and in the LDT

PPN is a heterogeneous structure whose specific functions are being difficult to establish (Gut and Winn, 2016). However, a growing number of studies separately analyzing diverse paradigms in either the anterior or posterior PPN have been able to ascribe distinct roles to each of these two regions (Alderson et al., 2006, 2008; Wilson et al., 2009; Ros et al., 2010; Martinez-Gonzalez et al., 2012). Thus, self-administration of nicotine is changed by posterior but not anterior PPN lesions (Alderson et al., 2006), and the locomotor response to repeated nicotine is only altered after posterior PPN lesions (Alderson et al., 2008). Lesions of the anterior versus posterior PPN resulted in different electrophysiological effects on the firing of the cuneiform nucleus (Jin et al., 2016). In addition, low-frequency 25-Hz stimulation in the anterior PPN worsened gait, while stimulation of the posterior PPN improved gait (Gut and Winn, 2015). These data suggest that anterior and posterior portions of the PPN are associated with different brain circuits and behavioral processes. In this context, data about anatomical differences between these two regions are essential in order to better understand the reported functional segregation. An elegant stereological study demonstrated that the estimated density of the cholinergic subpopulation of the PPN was significantly higher in the posterior PPN whereas the GABAergic one was significantly higher in the anterior PPN (Mena-Segovia et al., 2009). These data were confirmed in the present work, and the latter was extended to each of the two cell subsets expressing a different GAD isoform. More importantly, our present data clearly show that the glutamatergic population in the PPN is also topographically distributed regarding the anterior–posterior dychotomy, as indicated by the statistically significant preferential distribution of VGlut2-positive cells in the caudal half of the nucleus. This finding is consistent with the higher density of VGlut2/calbindin double-positive neurons reported in the caudalmost segments of PPN in comparison to the rostral ones (Martinez-Gonzalez et al., 2012), and also with the presence of a higher number of glutamatergic cells in the pars compacta of PPN versus the pars dissipata reported earlier (Wang and Morales, 2009). This finding provides additional evidence of the topographical organization of the PPN (Martinez-Gonzalez et al., 2011) and reveals that each of its three main cell phenotypes is differentially distributed in the anterior versus posterior PPN. Furthermore, the preferential caudal distribution of glutamatergic cells supports the recently suggested distinction between a mainly inhibitory rostral PPN and a predominantly excitatory caudal one (Mena-Segovia and Bolam, 2017). This topographical anatomical and neurochemical organization of the PPN likely underlies both region-specific local neural networks and sets of projection neurons, partly accounting for the above mentioned functional segregation. For example, PPN neurons projecting to the STh and the gigantocellular nucleus – identified as cholinergic and largely glutamatergic – are preferentially located in the caudal PPN (Martinez-Gonzalez et al., 2014), where these two subpopulations are significantly more abundant. In addition, different roles have been ascribed to single cell phenotypes in the PPN in relation to the sleep/wake cycle. Thus, while glutamatergic neurons strongly promote wakefulness, cholinergic neurons suppress slow cortical rhythms (Kroeger et al., 2017); thus, it is possible that both subpopulations work in concert to produce wakefulness (Kroeger et al., 2017). The fact that both subpopulations are preferentially located in the posterior PPN, and thus spatially close, supports potential neural and functional interactions between them.

To our knowledge, neither functional nor connectional studies in the LDT have investigated potential regional differences within the nucleus, probably due to the fact that its rostrocaudal extension is only ∼1.3 mm. The finding that the GABAergic subpopulation is preferentially located rostrally provides a first anatomical evidence of regional differences that will likely have connectional and functional correlates, thus far undetected.

### Glutamatergic Cophenotype of Cholinergic Neurons

The present results show that a small but significant percentage of PPN and LDT cholinergic cells (7.8 and 5.3%, respectively) also expressed Vglut2, and thus possess the molecular machinery to release glutamate. These percentages are consistent with a previous retrograde tracer study in which 5% of PPN neurons projecting to the parafascicular thalamic nucleus coexpressed Vglut2 and ChAT (Barroso-Chinea et al., 2011). A lower percentage of coexpression (2% in PPN and 1% in LDT) was reported previously (Wang and Morales, 2009), which may be due to the different quantitative method, the dual labeling protocol used, or to both. Earlier studies had illustrated the presence of glutamate in PPN cholinergic neurons in several species (Clements et al., 1991; Lavoie and Parent, 1994). The coexpression of the vesicular glutamate transporter reported here, however, adds to these data the potential to actually release glutamate.

There is increasing evidence of the coexistence of vesicular glutamate transporters in monoamine, cholinergic, and GABAergic neurons, raising questions about the role of neurons endowed with such a dual phenotype (El Mestikawy et al., 2011). Corelease of glutamate and acetylcholine has been reported in autaptic connections from basal forebrain cholinergic neurons in microculture (Allen et al., 2006; Huh et al., 2008), consistent with the coexistence of Vglut1 and/or Vglut2 in a subset of these neurons both in acute dissociated cell preparations and brain slices (Sotty et al., 2003; Danik et al., 2005). Corelease has also been reported in some spinal interneurons in the Xenopus tadpole, in neonatal and adult mouse spinal motoneurons (Li et al., 2004; Mentis et al., 2005; Nishimaru et al., 2005; Lamotte d’Incamps et al., 2017), in striatal cholinergic interneurons (Higley et al., 2011), and in projection neurons of the medial habenula (Ren et al., 2011).

Although electron microscopic studies have failed to reveal dually ChAT- and Vglut2-positive axon terminals in several thalamic nuclei innervated by the PPN and LDT (Parent and Descarries, 2008), ultrastructural studies in the entopeduncular nucleus and STh have shown the colocalization of ChAT and glutamate immunoreactivities in axon terminals, arising most likely from the mesopontine tegmentum (Clarke et al., 1996, 1997). The new data on ChAT and Vglut2 coexpression support a possible though minor corelease of acetylcholine and glutamate from PPN and/or LDT axon terminals. The ability to generate fast excitatory postsynaptic currents through the corelease of glutamate would likely confer this subset of dual cholinergic/glutamatergic cells a different electrophysiological profile to that of single cholinergic neurons. Whether any of the electrophysiological subtypes observed in the PPN (Steriade et al., 1990; Takakusaki et al., 1996, 1997; Mena-Segovia et al., 2008) corresponds to dual ChAT- and Vglut2-positive neurons remains to be analyzed.

## Author Contributions

EL, IH, MA, and EM: design of the study. EL and IH: experimental execution. EL, MA, and EM: data analysis and writing the manuscript.

## Conflict of Interest Statement

The authors declare that the research was conducted in the absence of any commercial or financial relationships that could be construed as a potential conflict of interest.

## Footnotes

1 https://www.zeiss.com/microscopy/int/products/microscope-software/zen-lite.html

## Abbreviations

ABC: avidin–biotin peroxidase complex
BSA: bovine serum albumin
ChAT: choline acetyl transferase
DAB: diaminobenzidine tetrahydrochloride
DEPC: diethyl propyl carbonate
GABA: gamma-aminobutyric acid
GAD: glutamic acid decarboxylase
HRP: horseradish peroxidase
ir: immunoreactive
ISH: fluorescent *in situ* hybridization
LDT: laterodorsal tegmental nucleus
PB: phosphate buffer
PBS: phosphate buffered saline
PPN: pedunculopontine tegmental nucleus
RT: room temperature
scp: superior cerebellar peduncle
SSC: standard sodium citrate
STh: subthalamic nucleus
TB: Tris-hydrochloric acid buffer
TN: Tris-hydrochloric acid-sodium chloride
TNT: Tris hydrochloric acid sodium chloride tween
TS: Tris saline
TSA: tyramide signal amplification
Vglut2: vesicular glutamate transporter 2
V: volume
