# Supplementary figures and images for: Stereological Estimates of Glutamatergic, GABAergic, and Cholinergic Neurons in the Pedunculopontine and Laterodorsal Tegmental Nuclei in the Rat
Source: Front Neuroanat. 2018 May 11;12:34. doi: 10.3389/fnana.2018.00034 (PMC5958217; doi:10.3389/fnana.2018.00034)

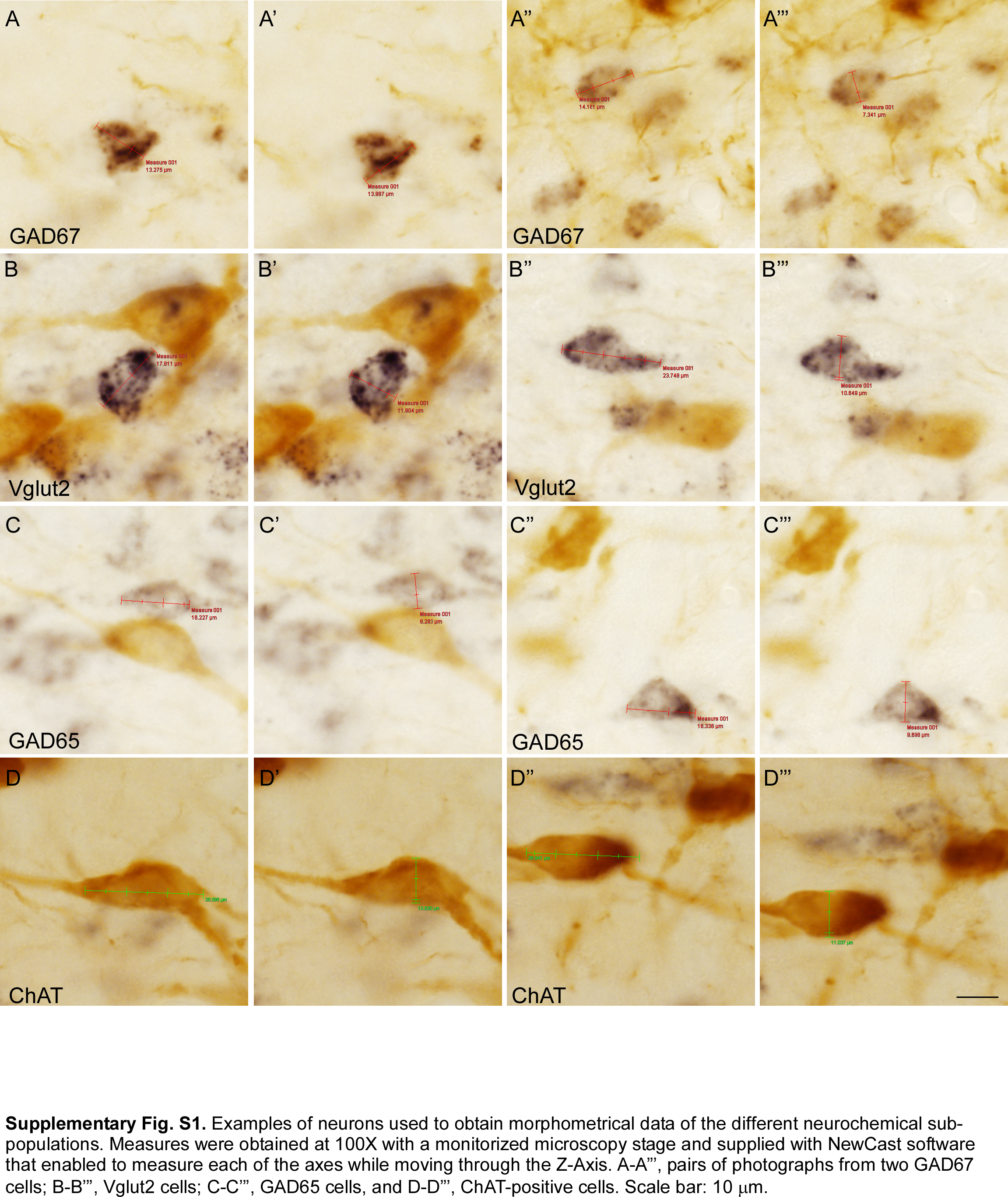

Supplement: Supplementary file 1 [file Image_1.TIF]

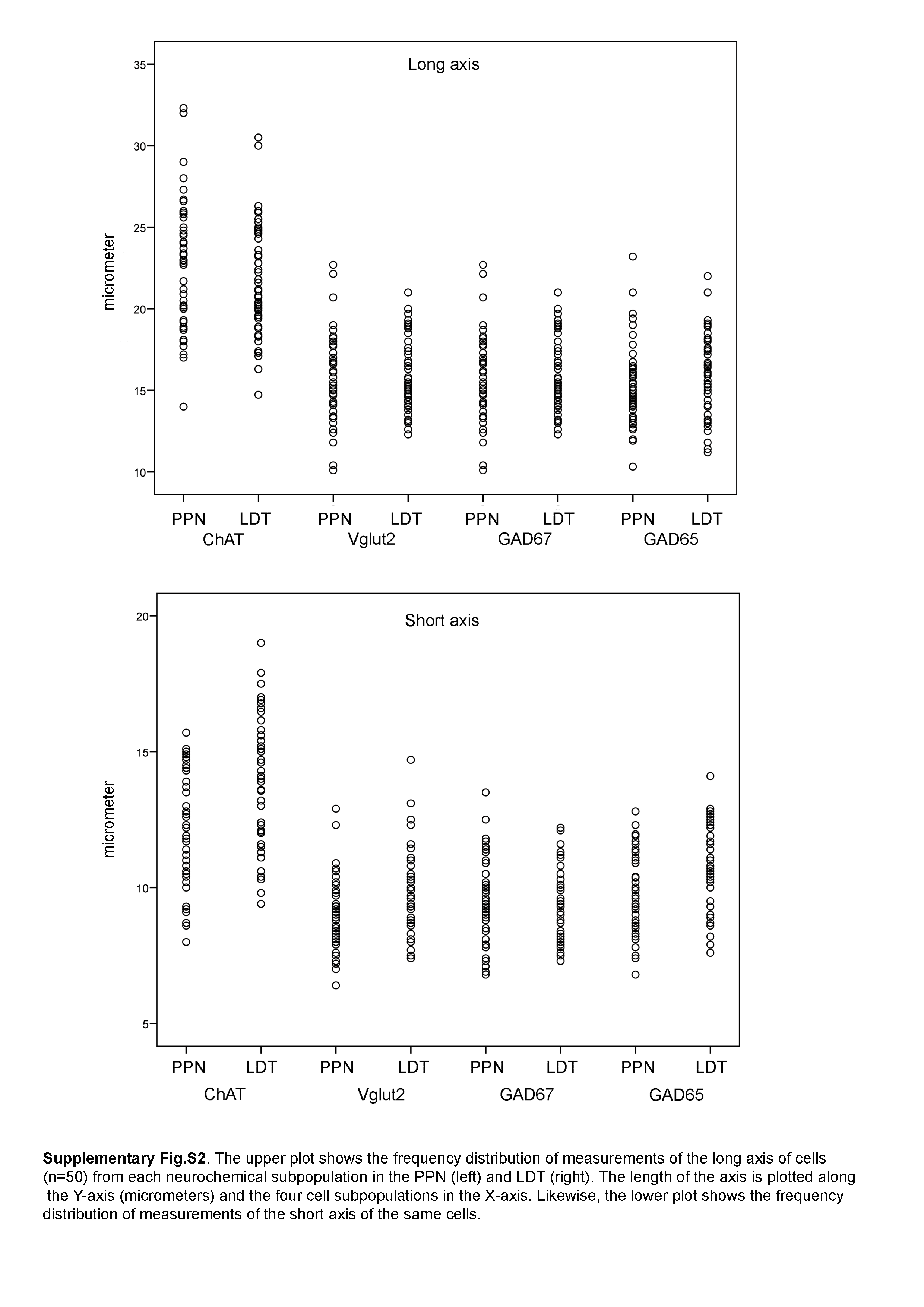

Supplement: Supplementary file 2 [file Image_2.TIF]

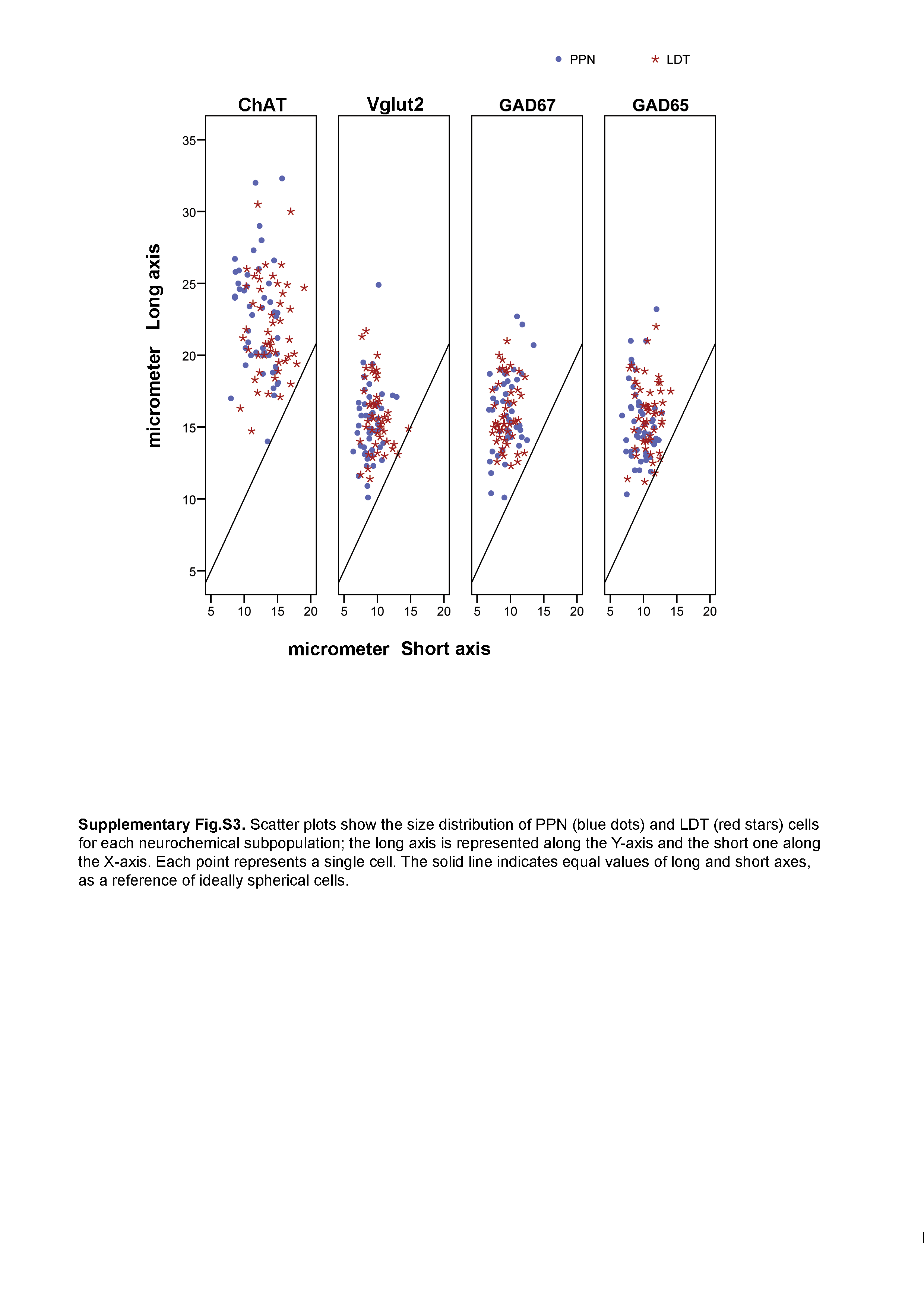

Supplement: Supplementary file 3 [file Image_3.TIF]
